# Etiopathogenesis of Immune‐Mediated Necrotizing Myopathy: A Comprehensive Review of Recent Advances

**DOI:** 10.1002/cns.70931

**Published:** 2026-05-15

**Authors:** Chang Gao, Wenli Li, Qingyan Liu, Guochun Wang, Qinglin Peng

**Affiliations:** ^1^ Department of Rheumatology, Key Lab of Myositis China‐Japan Friendship Hospital Beijing China

**Keywords:** autoantibodies, complement activation, idiopathic inflammatory myopathies, immune‐mediated necrotizing myopathy, mitochondrial dysfunction, regulated cell death

## Abstract

**Background:**

Immune‐mediated necrotizing myopathy (IMNM) is a rare autoimmune disease characterized by prominent muscle involvement and usually associated with serum autoantibodies targeting signal recognition particle (SRP) or 3‐hydroxy‐3‐methylglutaryl‐CoA reductase (HMGCR). Although the clinical manifestations, histopathological features, and disease classification of IMNM are well established, its etiopathogenesis remains incompletely understood.

**Objectives:**

This review aims to provide an up‐to‐date overview on the pathogenesis of IMNM.

**Results:**

Recent studies have implicated genetic susceptibility, autoantibodies, aberrant activation of immune cells and inflammatory mediators, and dysregulation of regulated cell death pathways in mediating tissue damage of IMNM. Anti‐SRP and anti‐HMGCR autoantibodies are pathognomonic for IMNM, and passive transfer animal models have confirmed that these autoantibodies directly mediate muscle damage, with complement activation playing a key role in this process. Dysregulation of immune cells including macrophages, T cells, and B cells, and inflammatory cytokines such as IFN‐γ, TNF‐α, and IL‐6 contributes to the inflammatory milieu and amplifies tissue injury. Furthermore, regulated cell death is involved in IMNM pathogenesis. Necroptosis may play an important role in myofiber damage in IMNM, with expression levels of RIPK3 and MLKL correlating with muscle disease severity. Pyroptosis and ER stress‐autophagy pathways have also been reported to participate in IMNM pathogenesis.

**Conclusion:**

IMNM is a distinct autoimmune myopathy driven by pathogenic anti‐SRP and anti‐HMGCR autoantibodies and multifaceted immune dysregulation involving immune cells, inflammatory cytokines, and multiple cell death pathways. Further elucidation of these mechanisms may facilitate the development of novel therapeutic strategies for IMNM.

## Graphical abstract

This review systematically clarifies that the core of IMNM begins with specific autoantibodies, which in turn trigger complement activation, immune cell infiltration, and a storm of inflammatory factors. Together, these processes induce regulatory cell death and mitochondrial dysfunction, ultimately leading to muscle fiber necrosis. Genetic factors and environmental triggers together form the basis of the pathogenesis of the disease.

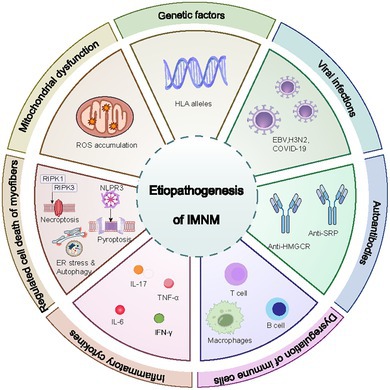

## 1 Introduction

Idiopathic inflammatory myopathies (IIMs) are a group of systemic autoimmune diseases that affect skeletal muscles and multiple organs, including the skin, lungs, gastrointestinal tract, and heart [1]. IIMs are a heterogeneous family with varying clinical, histopathological, and serological features and can be classified into major subgroups, including dermatomyositis (DM), polymyositis (PM), inclusion body myositis (IBM), antisynthetase syndrome (ASS), and immune‐mediated necrotizing myopathy (IMNM) [1]. IMNM is characterized by proximal muscle weakness, markedly elevated serum creatine kinase (CK) levels, and muscle pathology showing significant necrosis and regeneration while less lymphocytic infiltration [2, 3]. Although the epidemiological features of IMNM have not been fully elucidated, current research indicates that its incidence ranges from 0.6 to 0.83 per million person‐years, with a prevalence of 1.9 to 3.0 per million persons [4, 5].

The classification of IMNM has undergone significant evolution. In the early 20th century, IMNM was not considered a distinct myopathy, but was instead classified as PM or DM [6]. The identification of anti‐signal recognition particle (SRP) antibodies led researchers to determine distinctive pathological features of IMNM—extensive myofiber necrosis and regeneration with sparse inflammatory cell infiltration [7, 8]. Building on these findings, the European Neuromuscular Centre (ENMC) defined IMNM as a separate subtype of IIM in 2004 [9]. The subsequent discovery of anti‐3‐hydroxy‐3‐methylglutaryl‐CoA reductase (HMGCR) antibodies in 2010 expanded the classification of IMNM and provided a novel diagnostic marker [10]. Emerging research indicates that IMNM can be further divided into three subgroups: anti‐SRP‐positive, anti‐HMGCR‐positive, and seronegative [11].

**FIGURE 1:**
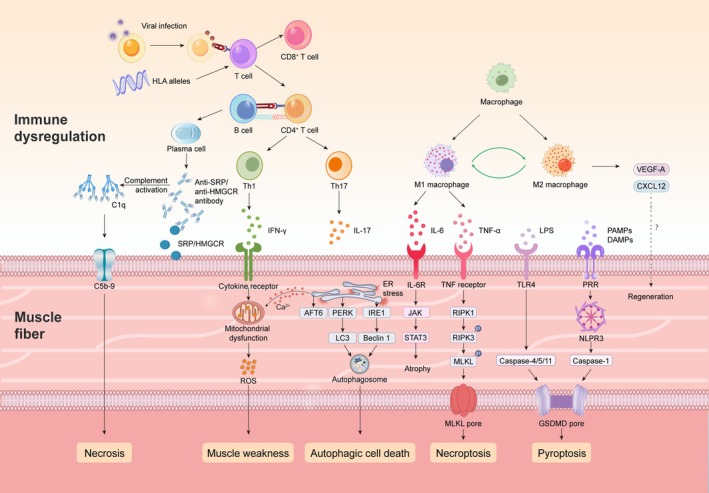
Implicated processes in the etiopathogenesis of IMNM. Environmental factors, including viral infections and genetic susceptibility, particularly specific HLA alleles, may disrupt autoimmune tolerance, resulting in abnormal activation of autoimmune responses against SRP or HMGCR. Autoreactive CD4^+^ T cells polarized toward Th1/Th17 phenotypes infiltrate muscle tissue, secreting proinflammatory cytokines (IFN‐γ and IL‐17) that both amplify local inflammation and activate B cells. Anti‐SRP/HMGCR antibodies bind to their target antigens on myofibers, activating the complement cascade and forming membrane attack complexes (C5b‐9), which directly induce sarcolemmal damage and necrosis. Meanwhile, IFN‐γ further disrupts mitochondrial function, triggering excessive ROS production that culminates in oxidative damage, metabolic dysfunction, and ultimately myofiber degeneration and muscle weakness. While M1 macrophages dominate the inflammatory milieu by releasing proinflammatory cytokines that exacerbate tissue injury, M2 macrophages may play a compensatory role in tissue repair. Pyroptosis may also exacerbate muscle damage through both canonical (NLRP3/caspase‐1/GSDMD) and noncanonical (LPS/caspase‐4/5/11‐GSDMD) pathways. ER stress, mediated by the PERK/IRE1/ATF6‐dependent unfolded protein response (UPR), not only promotes inflammation and apoptosis but also upregulates autophagy‐related genes, initiating autophagosome formation. Necroptosis has also been implicated in muscle fiber death via the TNF/RIP1‐RIP3‐MLKL axis, contributing further to programmed myofiber necrosis and amplifying inflammation.

Although the pathogenesis of IMNM is not yet fully understood, recent studies have highlighted the role of genetic, environmental, immune, and non‐immune mechanisms in its pathophysiology (Figure 1). This review aims to comprehensively discuss the contribution of genetic risks, environmental factors, and the role of autoantibodies, immune cells, inflammatory mediators, and regulated cell death mechanisms in mediating the muscle pathology of IMNM.

## 2 Clinical Features and Histopathological Findings of IMNM

### 2.1 Muscular Manifestations

Over two‐thirds of patients with IMNM typically present with an acute or subacute (< 6 months) onset, while one‐fourth exhibit insidious onset and a longer disease course (> 12 months) [12, 13]. Notably, patients with juvenile IMNM may present with slowly progressive proximal muscle weakness, which can lead to a misdiagnosis of muscular dystrophy [14, 15].

Approximately 80%–90% of patients with IMNM develop significant proximal muscle weakness early in the disease course, typically presenting with difficulty squatting and limited arm lifting [16, 17, 18]. However, some patients may also experience distal muscle weakness [19, 20]. Muscle weakness in IMNM is generally bilateral and symmetric; however, an estimated 10%–20% of patients exhibit asymmetric weakness [13, 16, 21]. Markedly elevated serum CK levels are a significant feature of IMNM, often exceeding 10–30 times the upper normal limit [3, 12, 13]. Magnetic resonance imaging (MRI) shows hyperintensity on T2‐weighted and STIR sequences, indicating edema and inflammation, while electromyography (EMG) reveals small, short‐duration, polyphasic motor unit potentials and increased muscle irritability [22].

### 2.2 Extramuscular Manifestations

Although IMNM primarily presents with muscle tissue inflammation and damage, it is also linked to various extramuscular manifestations, including interstitial lung disease (ILD), myocardial involvement, skin rash, dysphagia, arthritis, fever, and Raynaud phenomenon [3, 23].

ILD is more frequent in anti‐SRP‐positive IMNM (13%–45%) than in anti‐HMGCR‐positive cases (< 5%) [16, 24, 25]. Unlike ASS and anti‐MDA5‐positive DM, ILD in IMNM patients is typically mild [16, 24]. Myocardial involvement is characterized by arrhythmias and left ventricular diastolic dysfunction [26, 27]. Although cardiac involvement was initially reported in 2%–40% of anti‐SRP‐positive IMNM cases [1, 16, 28], recent studies confirmed its occurrence in patients with anti‐HMGCR‐positive IMNM (30.6%) [29], and seronegative IMNM (68.8%) [30]. Notably, recent studies report a considerable prevalence (15%–56%) of cutaneous manifestations in IMNM [31, 32, 33]. Dysphagia from oropharyngeal/esophageal involvement may present as an initial symptom of IMNM and progress rapidly [17, 34, 35, 36].

### 2.3 Histopathological Findings

Muscle biopsy is a vital diagnostic tool for seronegative and atypical IMNM. Myofiber necrosis was observed in over 90% of patients with IMNM to varying degrees, typically exhibiting diffuse distribution with occasional perimysial clustering [37, 38, 39, 40]. Regenerating muscle cells were observed in two‐thirds of patients with IMNM and also diffusely distributed [32, 41]. Necrotizing and regenerating myofibers were more frequent in anti‐SRP‐positive IMNM than in anti‐HMGCR‐positive IMNM [37, 39, 40, 42].

Inflammatory infiltrates in IMNM are predominantly composed of endomysial CD68‐positive macrophages in over 80% of cases [39, 43]. CD4‐positive T cells are present in over half of patients, located in the endomysium and perivascular regions, while CD8‐positive T cells are sparse and lack granzyme B expression [37, 39]. A key histological feature is the upregulated expression of MHC‐I in blood vessels and non‐necrotic fibers, showing scattered patterns in anti‐SRP‐positive IMNM versus aggregated or diffuse distribution in anti‐HMGCR‐positive variants, while MHC‐II expression is minimal [13, 43, 44, 45]. The deposition of C5b‐9, a diagnostic hallmark, correlates with necrotic muscle fibers and predominantly localizes to the sarcolemma of non‐necrotic myofibers in a scattered distribution [3, 13, 18, 39].

P62 is an autophagy‐related protein that mediates the degradation of ubiquitinated proteins [46]. In IMNM muscle biopsies, the expression of p62 was positive, showing a diffuse, finely dotted, and homogeneous pattern within the sarcoplasm in scattered necrotic fibers [47, 48]. In contrast, myxovirus resistance protein A (MxA), an activation marker of the type I interferon (IFN‐1) pathway, is rarely expressed (< 2%) in IMNM [49, 50, 51]. Given the much higher frequency of MxA positivity observed in DM [52], MxA has great potential to serve as a differential diagnostic marker for IMNM from DM.

## 3 Disease Subtypes Classified Based on Autoantibodies Against SRP and HMGCR

### 3.1 Anti‐SRP‐Positive IMNM

Anti‐SRP autoantibodies were first discovered in 1986 in the sera of patients with PM [8]. The target antigen of the anti‐SRP antibody is a universal ribonucleoprotein complex consisting of six polypeptide chains with molecular weights of 72, 68, 54, 19, 14, and 9 kDa, as well as a 7SL RNA molecule [53, 54]. This complex plays a crucial role in protein synthesis by ensuring nascent peptides are accurately targeted to the endoplasmic reticulum (ER) for folding, modification, and eventual secretion [55, 56].

The prevalence of anti‐SRP‐positive IMNM ranges from 5% to 18% among patients with IIMs, with a higher prevalence in Asian populations than in Caucasians [13, 57]. This disease primarily affects females aged 40–50 years [12, 58], and constitutes 2%–5% of juvenile IIMs [59]. Compared to anti‐HMGCR‐positive IMNM, the anti‐SRP‐positive patients are characterized by more severe muscle symptoms, including elevated CK levels, pronounced weakness, and extensive myofiber necrosis [13, 17].

### 3.2 Anti‐HMGCR‐Positive IMNM

In 2010, Christopher‐Stine et al. first identified autoantibodies against 200 kDa and 100 kDa proteins in patients with IMNM using immunoprecipitation, which were termed anti‐P200/100 antibodies [10]. The following year, the same research group confirmed that the 100 kDa target antigen was HMGCR [60], the enzyme responsible for the HMG‐CoA‐to‐mevalonate conversion in cholesterol synthesis and the target inhibited by statins [61, 62]. Anti‐HMGCR‐positive IMNM occurs in 6%–12% of patients with IIMs [60, 63]. In juvenile IIMs, anti‐HMGCR‐positive IMNM is rare, with a prevalence of approximately 1%, and is often severe and refractory to treatment [64].

It has been hypothesized that statins trigger the development of anti‐HMGCR‐positive IMNM. The prevalence of statin exposure in patients with anti‐HMGCR‐positive IMNM varies significantly across populations, with a frequency of 14% in Asia [65], 44.4% in Europe [63], and 65% in the United States [60]. Notably, statin‐naïve patients are distinctly younger, more frequently of non‐white ethnicity, and more commonly present with dysphagia [66]. Interestingly, dietary or environmental exposures other than strictly statin medications may also be associated with anti‐HMGCR antibody production and IMNM [67].

### 3.3 Seronegative IMNM

Approximately 20% of patients with IMNM are seronegative [11, 68], and their demographic characteristics and clinical manifestations are similar to those of patients with seropositive IMNM [12, 30]. However, seronegative IMNM is associated with a higher frequency of concomitant connective tissue diseases compared to seropositive IMNM [68, 69]. Several studies have reported that most patients with seronegative IMNM achieve a satisfactory response after immunosuppressive therapy [30, 68].

## 4 Immunopathogenesis

### 4.1 Genetic Factors

Previous studies showed that genetic risk factors for IIMs mainly lie within the human leukocyte antigen (HLA) region [70, 71]. Similarly, several studies also found that specific HLA alleles are significantly associated with an increased susceptibility to IMNM [72, 73].

The association between anti‐SRP‐positive IMNM and HLA genes appears to be ethnicity‐specific. Among Asian populations, especially in Japan and Korea, a strong association has been established between anti‐SRP‐positive IMNM and the HLA‐DRB1*14:03 and HLA‐DRB1*08:03 alleles [74, 75]. In contrast, no statistically significant association has been found in Caucasians [76].

Currently, most genetic data associated with anti‐HMGCR‐positive IMNM have been obtained from small cohorts. The HLA‐DRB1*11:01 allele has been strongly associated with anti‐HMGCR‐positive IMNM in adults, with a significantly stronger correlation when carriers are exposed to statins [73, 77]. Additionally, HLA‐DRB1*07:01 has been associated with the disease in juvenile patients [78]. Interestingly, one small study found that the frequency of HLA‐DRB1*11:01, but not HLA‐DRB1*07:01, was significantly elevated in statin‐naïve children and young adults with anti‐HMGCR‐positive IMNM who experienced a chronic disease course that mimicked limb‐girdle muscular dystrophy [79]. Large‐scale international multicenter studies are required to clarify the genetic basis of IMNM and their specific autoantibodies.

### 4.2 Viral Infections

Viral infection has been proposed as a potential trigger for IMNM. Leff et al. observed seasonal variation in the incidence of anti‐SRP‐positive IMNM, with a high occurrence during autumn and winter, seasons where viral infections are highly prevalent [80]. Emerging case reports have shown a link between IMNM and Epstein–Barr virus (EBV) infections [81], influenza A (H3N2) [82], and dengue infection [83]. Furthermore, during the COVID‐19 pandemic, case reports have highlighted disease recurrence in patients with anti‐HMGCR‐positive IMNM following SARS‐CoV‐2 infection and the onset of IMNM post‐vaccination [84, 85]. Notably, Aschman et al. demonstrated that skeletal muscle from severe COVID‐19 fatalities exhibited myositis, with early MHC‐I and late MHC‐II upregulation, plus significant infiltration of CD45, CD8, and NK cells [86]. However, the inflammatory changes observed in these patients may reflect a broader systemic response to severe viral infection, rather than the specific pathology of IMNM [87]. Moreover, the lack of MHC‐I and lymphocyte subset co‐staining undermines the claim of T‐cell‐ or macrophage‐mediated myocytotoxicity and fails to establish a direct causal link between SARS‐CoV‐2 and myositis [87]. Taken together, these studies provide insights into the contributing role of infection in the development of IMNM; however, more rigorous evidence is needed to establish a causal link between infection and IMNM.

### 4.3 Autoantibodies

Anti‐SRP and anti‐HMGCR autoantibodies serve as highly specific serological markers for IMNM and constitute essential components for disease diagnosis and classification. Moreover, strong correlations between autoantibody titers and disease activity have been reported in patients with IMNM [13, 63, 88]. A recent study has directly demonstrated immunoglobulin G deposition within the cytoplasm of myofibers in patients with these autoantibodies, confirming their presence at the site of disease pathology [89]. In addition, intravenous immunoglobulin (IVIG) has been reported to be an effective therapy for IMNM [11, 90, 91, 92, 93]. The potential mechanisms underlying the therapeutic effects of IVIG include neutralization and accelerated clearance of pathogenic autoantibodies, reduced complement activation and membrane attack complex deposition on muscle fibers and capillaries, decreased expression of adhesion molecules and cytokine production, and suppression of pathogenic T‐cell activation [94]. Together with clinical observations that plasma exchange, which potentially removes these autoantibodies, leads to measurable improvements in muscle strength [95], these findings suggest that autoantibodies are involved in the pathogenesis of IMNM.

To test the hypothesis that anti‐SRP and anti‐HMGCR antibodies contribute to muscle damage in IMNM, Rojana et al. first used high‐titer anti‐SRP‐positive IMNM serum to stimulate human myoblasts and reported significantly reduced cell viability [96]. Furthermore, Arouche et al. conducted in vitro studies demonstrating that purified IgG from patients with anti‐SRP and HMGCR‐positive IMNM induced significant muscle fiber atrophy and increased the expression of MAFbx and TRIM63 [97]. This antibody‐mediated damage, which reduced IL‐4 and IL‐13 production and impaired muscle regeneration, could be reversed through cytokine reintroduction, which restored myotube formation [97].

A murine model also demonstrated the pathogenicity of autoantibodies against SRP and HMGCR in the context of muscle damage. By passively transferring anti‐SRP or anti‐HMGCR IgGs to wild‐type mice, Bergua et al. found that recipient mice developed significant muscle necrosis and reduced muscle strength within 7 days [98]. Moreover, mice injected with anti‐SRP‐positive IgG exhibited more severe muscle weakness than those injected with anti‐HMGCR‐positive IgG, which is consistent with clinical observations in patients with IMNM [98]. Notably, the decrease in muscle strength was significantly ameliorated when these autoantibodies were injected into C3 complement fragment (C3^−/−^)‐deficient mice [98]. Upon supplementation with human complement, the degree of muscle damage in the mice was significantly aggravated [98]. Overall, these findings highlight the pathogenic role of anti‐SRP and anti‐HMGCR antibodies in IMNM‐related muscle damage and suggest that complement activation may be a key factor in mediating autoantibody‐induced muscle pathology in IMNM. Based on these findings, Julien et al. treated a humanized mouse model of IMNM with zilucoplan, a complement C5 inhibitor, which demonstrated prophylactic effects by reducing C5b‐9 deposition, alleviating muscle weakness, and promoting muscle fiber regeneration [99]. However, a multicenter phase II clinical trial showed that zilucoplan demonstrated no significant efficacy in either CK levels or in alleviating clinical symptoms in adult patients with anti‐SRP or anti‐HMGCR‐positive IMNM [100]. Therefore, the role of the complement system in the pathological mechanism of IMNM may be complex and warrants further investigations.

### 4.4 Dysregulation of Immune Cells and Inflammatory Mediators

#### 4.4.1 Macrophages

Macrophage infiltration is a prominent feature in muscle biopsy of patients of IMNM [3, 96]. Depending on the immune environment [101, 102, 103], macrophages can differentiate into distinct functional subtypes, classically activated M1 macrophages (Table 1) and alternatively activated M2 macrophages. Preuß et al. demonstrated dominant M1 responses associated with pro‐inflammatory cytokine production in a cohort of 16 IMNM patients [104], while Chung et al. reported predominantly M2 polarization in anti‐HMGCR‐positive IMNM in a study involving 18 patients [107]. In addition, Lia et al. demonstrated that both M1 and M2 macrophages infiltrated the perivascular endomysium and expressed angiogenic factors, such as VEGF‐A and CXCL12, in anti‐HMGCR‐positive IMNM [108]. Importantly, the density of VEGF‐A^+^ M2 macrophages correlates with angiogenesis, highlighting the role of these cells in promoting muscle regeneration [108].

**TABLE 1:** Implications of immune cell subsets in the pathogeneses of IMNM.

	Pathogenic role	Clinical evidence	Refs.
M1 macrophages	M1 macrophages are activated by the TLR/NF‐κB pathway and secrete pro‐inflammatory factors, which directly damage muscle cell membranes	M1 macrophage infiltration was observed in muscle biopsies from patients with IMNM	[103, 104]
CD226^+^ T cell	CD226, an activating receptor of the Ig superfamily expressed on CD8^+^ T and NK cells, binds CD155 on muscle fibers and enhances cytotoxicity and IFN‐γ/TNF‐α production, driving muscle necrosis in IMNM	CD226^+^ T cells are significantly elevated in IMNM muscle tissue, correlate with disease severity, and decrease after immunosuppressive treatment	[105]
PD‐1^+^ T cell	PD‐1^+^CD8^+^ T cells damage muscles by releasing cytotoxic molecules (perforin/granzyme B). Normally, IFNγ‐induced PD‐L1 on muscle cells inhibits these T cells, but this protective mechanism fails in myositis	PD‐1^+^CD8^+^ T cells with elevated perforin/granzyme B infiltrate muscles and correlate with disease activity	[106]

#### 4.4.2 T Cells

Studies have implicated T cells as pivotal orchestrators driving muscle inflammation and damage in IMNM. Most recently, Tiniakou et al. demonstrated the presence of HMGCR‐reactive CD4^+^ T cells skewed toward a Th1‐Th17 phenotype in anti‐HMGCR‐positive IMNM, suggesting their active role in pathogenesis [109]. Knauss et al. further found that most CD8^+^ T cells in muscle biopsies of patients with IMNM were PD1‐positive [110], which is in line with earlier findings that T cells exhibit limited cytotoxicity in IMNM muscle owing to the absence of granzyme B‐positive CD8^+^ T cells [39]. In contrast, Sasak et al. used chemically induced myositis (CIM) model to reveal that peripheral PD‐1^+^ T cells in patients with active‐phase PM/DM present with an effector phenotype and found that PD‐L1‐deficient mice developed more severe myositis with prominent infiltration of PD‐1^+^CD8^+^ cells expressing cytolytic molecules than wild‐type mice, thus suggesting a pathogenic role of PD‐1^+^CD8^+^ cells in this myositis model [106]. The potential dual nature of PD‐1^+^ T cells highlights the complexity of the immune response to IMNM and warrants further investigation.

Intriguingly, Li et al. found that CD155‐CD226‐mediated stimulatory signaling was much stronger than CD155‐TIGIT‐mediated co‐inhibitory signaling in the muscle microenvironment of DM and IMNM [105]. Moreover, the CD155‐CD226 axis was strongly associated with disease activity and degree of muscle damage, potentially leading to overactivation of effector T cells and persistent inflammation [105]. These data highlight the complex interplay between T cells and the muscle microenvironment in IMNM.

#### 4.4.3 B Cells

Given the established pathogenic roles of anti‐SRP and anti‐HMGCR autoantibodies, B cells that produce these antibodies are increasingly recognized as potential contributors to disease pathogenesis. B cell activating factor (BAFF), an important factor in B cell survival and maturation, was found to be overexpressed in muscle fibers of anti‐SRP‐positive IMNM [38, 111]. In addition, expression of the BAFF receptor (BAFF‐R) was significantly higher in refractory patients than in non‐refractory patients, suggesting that BAFF‐mediated B cell activation may be involved in muscle fiber injury [38]. Intriguingly, the therapeutic efficacy of belimumab, a human monoclonal antibody targeting BAFF, has been reported in a case study of anti‐SRP‐positive IMNM [112].

B cell maturation antigen (BCMA) plays a crucial role in B cell survival and humoral immunity regulation [113]. BCMA‐targeted chimeric antigen receptor T (CAR‐T) cell therapy is a promising approach for autoimmune diseases. Recent studies have documented the successful use of BCMA‐ or CD19‐targeted CAR‐T cell therapy in two individual cases of refractory anti‐SRP‐positive IMNM, demonstrating significant clinical efficacy and a good safety profile [114, 115]. Further studies with larger cohorts are needed to better understand the effect of CAR‐T cells on B cells in autoimmune diseases and their modulation of the immune system.

#### 4.4.4 Inflammatory Cytokines

Inflammatory cytokines have also been implicated in exacerbating muscle damage by modulating the immune response and promoting inflammation in IMNM. For instance, IFN‐γ contributes to this process by activating the JAK–STAT pathway, which upregulates interferon‐stimulated genes and promotes inflammatory cell infiltration [116, 117]. Similarly, TNF‐α contributes to muscle atrophy in IMNM by activating the NF‐κB pathway and upregulating the expression of MuRF1 and MAFbx. Its pathogenic role is further supported by elevated serum levels that correlate with disease activity [97, 118].

Oda et al. demonstrated that IP‐10, MIP‐1α, and MCP‐1 levels were correlated with serum CK levels and significantly decreased after immunosuppressive therapy, indicating the involvement of these cytokines in the pathogenesis of IMNM by activating macrophages [119]. Among them, MCP‐1 is significantly associated with inflammatory infiltration and myofiber necrosis in IMNM biopsies [120]. In vitro studies revealed that the increase in MCP‐1 expression in human myoblasts was induced by the IL‐6/sIL‐6R complex via the STAT3 pathway, which may be mechanistically caused by enriched phospho‐STAT3 in the MCP‐1 promoter region [120]. IL‐6 is a pivotal mediator in IMNM. It activates the JAK/STAT3 pathway, which suppresses satellite cell function, and its level correlates with disease activity [121, 122]. The clinical significance of IL‐6 is supported by its correlation with IMNM disease activity and the treatment response to tocilizumab in refractory cases [123]. These findings underscore the importance of inflammatory mediators in IMNM progression (Table 2).

**TABLE 2:** Implications of inflammatory mediators in the pathogenesis of IMNM.

	Pathogenic role	Clinical evidence	Refs.
IL‐6	IL‐6 activates the JAK/STAT3 pathway, suppressing satellite cell expansion and impairing skeletal muscle development by interfering with growth hormone and IGF‐1 signaling	Serum IL‐6 concentrations correlate with disease activity. Tocilizumab, an anti‐IL‐6R monoclonal antibody, is effective in some refractory cases of IMNM	[121, 122, 123]
IL‐17	IL‐17 synergizes with IL‐6 to amplify autoantibody production, and activates fibroblasts—driving myofibrosis development	IL‐17 levels correlate with serum CK levels and disease duration in IMNM	[119]
IFN‐γ	IFN‐γ induces ISG expression by activating the JAK–STAT signaling pathway, promotes infiltration of inflammatory cells and damage to muscle cells	IFN‐γ‐induced gene expression levels are positively correlated with the expression of inflammatory cells and muscle regeneration‐related genes in IMNM	[116, 117]
TNF‐α	TNF‐α promotes skeletal muscle atrophy by activating NF‐κB, leading to upregulation of MuRF1 and MAFbx	The serum level of TNF‐α in IMNM is significantly increased and is related to disease activity	[97, 118]
CXCL12	CXCL12 promotes myosatellite cell migration and differentiation via the CXCR7 receptor in endothelial cells	CXCL12 was significantly overexpressed in the muscle tissues of patients with anti‐HMGCR‐positive IMNM and was significantly positively correlated with capillary density (CD31^+^)	[108]
BAFF	BAFF promotes the survival of B cells through BAFF‐R, activates the non‐canonical NF‐κB pathway of TRAF3/NIK/IKK1, and promotes the survival of mature B cells, thereby continuously producing autoantibodies	Belimumab, a BAFF/APRIL inhibitor, has shown efficacy in individual IMNM cases	[111, 112]

### 4.5 Regulated Cell Death of Myofibers

#### 4.5.1 Necroptosis

Necroptosis is a regulated form of necrotic cell death, mediated by the sequential activation of RIPK1, RIPK3, and mixed‐lineage kinase domain‐like (MLKLs), ultimately leading to plasma membrane rupture and release of inflammatory cellular contents [124, 125].

Several studies have demonstrated that activation of necroptosis in IIMs, including INMN, may contribute to myofiber damage. Peng et al. demonstrated that the expression of key factors mediating necroptosis, including RIPK3 and MLKL, was highly upregulated and correlated with the severity of muscle involvement in patients with IMNM and DM [126]. In vitro studies further demonstrated that C2C12 myocytes undergo necroptosis upon TNF‐α/z‐VAD stimulation, a process prevented by MLKL knockdown or necroptosis inhibitors [126]. Interestingly, a murine study utilizing a CIM mouse model of myositis also found that muscle inflammation and muscle necrosis were significantly decreased in both RIPK3^−/−^ and MLKL^−/−^ CIM mice [127]. Moreover, Necrostatin‐1s (Nec‐1s), a necroptosis inhibitor that targets RIPK1 kinase, significantly improved muscle strength and reduced muscle inflammation in both prophylactic and therapeutic treatments [127]. Furthermore, the same research group demonstrated that glucagon‐like peptide‐1 receptor agonists could inhibit myofiber necroptosis and recover muscle weakness in CIM mice, underscoring its potential as a novel therapy [128]. Based on these findings, necroptosis emerged as a potential therapeutic target for the treatment of myofiber damage in myositis, including IMNM.

#### 4.5.2 Pyroptosis

Pyroptosis, a regulated pro‐inflammatory cell death, can be triggered through multiple pathways involving different caspases and GSDMD [129]. Liu et al. demonstrated that upregulated glycolysis (particularly via PKM2) in the muscle tissue of patients with PM or DM activated the NLRP3 inflammasome, leading to GSDMD‐mediated pyroptosis in muscle cells [130]. Moreover, Ma et al. found that pyroptosis‐related factors, including caspase‐4/5/11, GSDMD, and NLRP3, were highly expressed in experimental autoimmune myositis (EAM) mice, suggesting overactivation of the non‐classical pyroptosis pathway in the pathogenesis of myositis [131]. Further studies are required to clarify the molecular pathways that regulate pyroptosis activation during the development and progression of IMNM.

#### 4.5.3 Endoplasmic Reticulum Stress and Autophagy

The endoplasmic reticulum (ER) is the primary site for protein synthesis. ER stress occurs when an accumulation of misfolded proteins exceeds the folding capacity of molecular chaperones like GRP78/BiP, leading to the activation of the unfolded protein response (UPR) [132, 133]. Interestingly, Preusse et al. found that the levels of molecules of the UPR pathway, including PERK, eIF2α, IRE1, ATF6, and BiP, were significantly increased in muscle biopsies of patients with IMNM [134].

Autophagy, a lysosomal degradation process essential for cellular homeostasis, is markedly upregulated in IMNM [135, 136]. Patients with IMNM showed significantly higher levels of autophagy markers LC3b and p62 in myofibers than those with other types of IIMs [136]. Fischer et al. revealed that key molecules mediating chaperone‐assisted selective autophagy (CASA), including BAG3, HSP70, and HSPB5, co‐localize with p62 within the muscle fibers of patients with IMNM [48], highlighting CASA involvement in protein homeostasis, cellular stress, and immune responses in the skeletal muscle of IMNM patients.

Importantly, autophagy is closely linked to ER stress, which can trigger autophagosome formation through UPR pathways and calcium‐mediated AMPK/mTORC1/ULK1 signaling, thereby facilitating clearance of misfolded proteins [137, 138]. A single‐center study involving 37 patients with IMNM found that activation of ER stress correlates with muscle weakness in IMNM, and interestingly, may be associated with multiple physiological and pathological processes, ranging from destruction to restoration in IIMs [139]. This underscores the multifaceted role of ER stress‐induced autophagy pathways in the pathophysiology of IMNM [139].

### 4.6 Mitochondrial Dysfunction

Mitochondria act as intracellular energy factories and are key factors in sustaining normal muscle cell function [140]. In muscle biopsies from patients with anti‐HMGCR‐positive IMNM, the accumulation of impaired mitochondria was observed, along with mitophagy‐related BNIP3 protein upregulation, implicating compromised mitophagy as a potential contributing factor to myofiber degeneration [141].

Mitochondrial dysfunction also leads to the accumulation of ROS, triggering oxidative stress. In an Icos^−/−^ NOD mouse model, mitochondrial defects and elevated ROS were observed, and intervention with ROS buffer therapy concurrently restored mitochondrial function and ameliorated inflammation [142]. Another study investigating the pathogenic role of anti‐SRP antibodies in mediating cardiac diastolic dysfunction found that in vivo passive transfer of total IgG from anti‐SRP‐positive patients to mice induced prominent left ventricular diastolic dysfunction with significantly increased cardiac ROS levels and altered mitochondrial integrity and function in mice [143]. These findings highlight the interplay among mitochondrial dysfunction, oxidative stress, and inflammation in the pathogenesis of IIMs, including IMNM.

## 5 Conclusion

IMNM has recently been recognized as a distinct disease entity characterized by muscle weakness, high CK levels, histopathological features with myofiber necrosis and regeneration with inflammatory infiltration, and the presence of anti‐SRP or anti‐HMGCR autoantibodies. With advances in research, the development of animal models established by passive transfer of human anti‐SRP or anti‐HMGCR IgGs to mice clearly demonstrates the pathogenicity of autoantibodies in mediating muscle damage in IMNM and provides a useful tool for further mechanistic studies. In addition, the complexity of etiology in IMNM may involve both immune and non‐immune mechanisms, highlighting the contributing role of dysregulation of macrophages, T cells, B cells, inflammatory cytokines, as well as regulated cell death in the pathogenesis of IMNM. Further investigation of disease pathophysiology would expand the current understanding of IMNM and aid in the development of new therapeutic strategies.

## Author Contributions

**Chang Gao:** writing – original draft, validation, formal analysis, visualization, software, methodology. **Wenli Li and Qingyan Liu:** data curation. **Guochun Wang:** investigation, resources. **Qinglin Peng:** methodology, writing – review and editing, funding acquisition, resources, project administration. All the authors have read and agreed with the published version of the manuscript.

## Funding

This work was supported by the Elite Medical Professionals Project of China‐Japan Friendship Hospital (No. ZRJY2024‐BJ04); the National High Level Hospital Clinical Research Funding (2025‐NHLHCRF‐JBGS‐A‐WZ‐18); the National Natural Science Foundation of China (82371810, 82572056).

## Ethics Statement

The authors have nothing to report.

## Consent

The authors have nothing to report.

## Conflicts of Interest

The authors declare no conflicts of interest.

## Data Availability

Data sharing not applicable to this article as no datasets were generated or analysed during the current study.
